# Physiological processes induced by different types of physical activity that either oppose or enhance postprandial glucose tolerance

**DOI:** 10.3389/fendo.2025.1601474

**Published:** 2025-07-04

**Authors:** Marc T. Hamilton, Deborah G. Hamilton, Theodore W. Zderic

**Affiliations:** ^1^ Department of Health and Human Performance, University of Houston, Houston, TX, United States; ^2^ Department of Biology and Biochemistry, University of Houston, Houston, TX, United States; ^3^ College of Optometry, University of Houston, Houston, TX, United States

**Keywords:** type 2 diabetes, impaired glucose tolerance, oral glucose tolerance, physical activity, glycogen, soleus, sedentary, exercise

## Abstract

Herein, we describe overlooked/misunderstood physiological processes (beyond contraction-induced skeletal muscle insulin sensitivity that is already well appreciated) that either oppose or enhance glucose tolerance during distinct types of acute physical activity. This includes multiple mechanisms both within and outside of muscle. We describe the processes and physiological principles to help explain why postprandial glucose tolerance is often not improved after acute bouts of exercise, or when interrupting prolonged sitting with either brief physical activity breaks or more prolonged standing. We also describe results from a specialized type of soleus muscle activity that is specifically well-geared to amplify and sustain oxidative muscle metabolism for long periods of time when sitting, with evidence that this has meaningful positive effects on systemic glucose and lipid regulation. Methods capable of elevating oxidative muscle metabolism could be advantageous to complement other lifestyle and pharmacological approaches whose mechanisms of action are limited to non-oxidative metabolic pathways. There is much potential need for inducing more oxidative muscle metabolism, because the entire musculature normally accounts for only about 15% of the oxidative metabolism of glucose when sitting inactive, despite being the body’s largest lean tissue mass. A clear understanding of the multiple integrative processes that either tend to attenuate or amplify blood glucose excursions in the postprandial period is significant, given the strong influence of glucose tolerance on healthy aging and prevention of multiple chronic diseases.

## Introduction

We describe our perspective of the physiological principles that determine the effects (or lack thereof) of different types and amounts of acute physical activity on postprandial glucose excursions. We summarize systemic responses in the minutes to hours after acute bouts of large muscle mass physical activity that tend to *oppose* glucose lowering in the subsequent postprandial period (1–8), despite evidence of increased insulin sensitivity in the recruited muscle mass (1, 7). We also provide a physiological perspective for why many studies have demonstrated the difficulty in causing large and significant reductions in postprandial glucose excursions when the duration of contractile activity throughout the postprandial period is potentially too brief (see **Supplementary Table** for a comprehensive annotated bibliography (1, 2, 4–6, 9–52). Additionally, we include studies that sometimes tested more prolonged standing (similar to using a standing desk) that also generally fail to improve postprandial blood glucose regulation (9–22). Finally, we discuss promising findings that more specifically tested the effects of a high local rate of oxidative metabolism targeted to the slow oxidative soleus muscle for a particular type of sustained contractile activity that causes potent metabolic improvements (49). We briefly describe how this local contractile activity at a sufficient metabolic rate can magnify the distinct biochemical phenotype of the soleus muscle, and this favors substrate utilization that improves blood glucose and blood lipid concentrations (49).

In order to expedite progress in developing improved lifestyle approaches to prevent complex chronic diseases and enhance health throughout aging, it is important to clearly describe some of the fundamental principles about muscle physiology. This article focuses only on highlighting several distinct processes that are important to glucose metabolism during the postprandial period, yet are frequently overlooked/misunderstood in the physical activity literature.

Quantifying the glucose excursion after ingesting a standard glucose load has proven to be an excellent test for understanding the specific biochemical processes important for preventing chronic disease and favoring healthier aging. Even in people who never progress to type 2 diabetes (T2D), the postprandial glucose responses to an oral glucose tolerance test (OGTT) are one of the strongest independent metabolic predictors of many of the same pathological conditions prevalent in patients living with T2D. Glucose responses during an OGTT that are frequently linked to aging-associated chronic disease include Alzheimer’s disease (53), neuropathies (54, 55), lipid disorders (56, 57), multiple cardiovascular conditions (57, 58), and more.

## Outlining four guiding physiology principles that should not be overlooked when describing glucose tolerance responses to acute physical activity

We suspect that, after a critical review, many people would be surprised that many well-controlled studies found that aerobic endurance exercise did not improve the subsequent glucose tolerance during an oral glucose tolerance test or carbohydrate load in a meal (1–6, 29, 39, 44, 50, 51, 59). Furthermore, standing during the postprandial period (9–15, 19, 21) or interrupting prolonged sitting with brief walking breaks (10, 16, 25–27, 29, 31, 32, 34, 37–39) frequently demonstrate no improvement in glucose tolerance (references with annotations in **Supplementary Table**). The scientific challenge we propose is to understand these and other key issues well enough to better optimize how to capitalize on the molecular machinery within skeletal muscle. We hypothesize that this has potential to change perspectives about how to engage in contractile activity within the postprandial period to better improve glucose tolerance. 

We present below four of the *most important, but frequently overlooked, fundamental muscle physiology principles* that we believe are required for a more clear understanding of potential solutions to improve the regulation of postprandial glucose. A complete list of references will be provided in subsequent subsections instead of here:

- the benefit of sustaining contractile activity for a sufficient duration to fully capitalize upon the potentially potent *direct effects of raising oxidative metabolism* on blood glucose regulation;
- an appreciation of the fact that there are opposing *systemic influences* induced during and after some modes of activity that can prevent glucose lowering, even when the recruited muscle is taking up more blood glucose;
- an understanding that the *phenotype of specific muscles recruited* impacts not only the fatigue response and thus the duration of activity, but also which types of metabolic substrates are utilized to generate ATP;
- the realization that blood glucose is generally not the dominant *carbohydrate* oxidized to fuel the working muscles, because muscle glycogen accounts for >70% to 90% of the carbohydrate utilization during a wide range of intensities (light-moderate intensity included) and types of physical activity (60–63).

These principles are intended to contribute to a paradigm that researchers and clinicians can use to test and understand physiological principles explaining why studies often report that glucose tolerance is not statistically improved by various types of muscular activity completed before (1–6, 29, 39, 44, 50, 51, 59) or during (9–16, 19, 21, 26, 27, 29–32, 34, 37–39, 41, 43) the postprandial period. We will frame this article largely around *recent advances in our driving goal of developing a specific type of targeted contractions to improve glucose and lipid regulation* (49, 64–68). Herein, we will describe the rationale for focusing on the potentially potent effects of sustaining oxidative muscle metabolism. This involves amplifying the effects of soleus muscle metabolism as much as possible instead of sitting inactive for prolonged periods.

## Results leading to four physiological principles important for glucose tolerance

### I. The *direct effects of acutely sustaining oxidative metabolism* in muscle above the low levels during inactivity are limited by the *duration* of contractile activity

**Figure 1:**
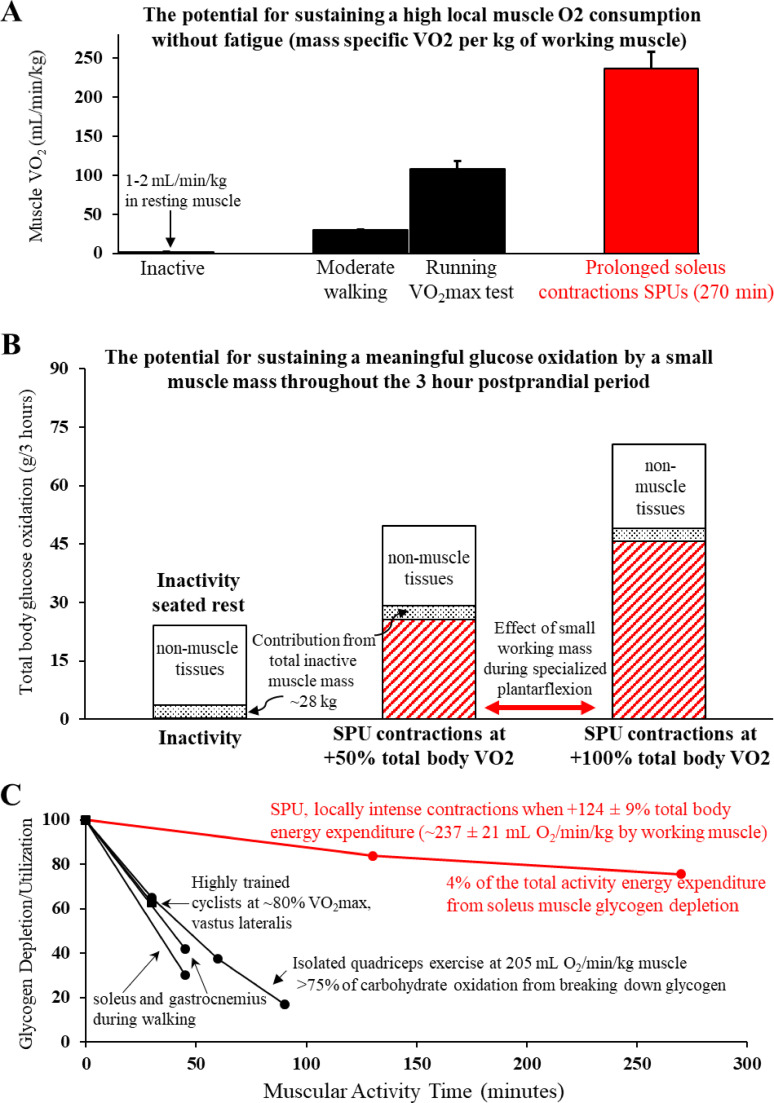
A unique type of locally intense, but fatigue-resistant contractile activity dominated by a slow-twitch oxidative muscle (**(A)** Soleus Push Up or SPU) amplifies oxidative muscle metabolism of the soleus throughout the postprandial period **(B)**, with relatively less reliance on intramuscular glycogen than other types of muscular activity **(C)**. **(A)** The SPU contractions can *sustain* a relatively high mass specific muscle VO_2._ The comparative estimates of large muscle mass activities were in the same volunteers that were tested for SPU contractions (49). **(B)** The calculated contribution of skeletal muscle tissue to total body glucose oxidation is enhanced by 2 levels of SPU contractions (49). Both the moderate intensity SPU (~100% increase in total body VO_2_) and also the light intensity SPU at half that level provided a meaningful process to *continuously* increase the impact of contracting muscle throughout the 3 hour postprandial OGTT. Note that while it is known skeletal muscle is not the dominant tissue for oxidative metabolism of blood glucose in the postprandial period (69) as illustrated also here, this singular action of SPU contractions by a small muscle mass alone is sufficient for muscle to dominate the whole body oxidative responses. **(C)** In comparison to other types of muscular activity that also demand a high oxygen demand by the working muscle, the SPU contractions (49) were found to cause relatively little glycogen depletion in the soleus compared to the vastus lateralis during intense cycle ergometry (70) or isolated quadricep muscle contractions (62), and both the soleus and gastrocnemius during a much larger muscle mass activity like brisk uphill walking (71). Panels **(A, B)** were adapted from (49).

In the absence of acute contractile activity, the oxygen consumption of muscle is relatively low at rest, averaging approximately 1–2 mL/min/kg (**Figure 1A**) when measured with arteriovenous balance method in the lower limbs (69, 72). This is low compared to other smaller lean tissues in vital organs (73), given that the total body oxygen consumption when sitting at rest to watch TV or do other common sedentary behaviors is about 3.5 mL/min/kg (74). Although the energy demand of skeletal muscle fibers can quickly increase 100 times or more above rest during acute contractile activity (75), *the often overlooked logical corollary of this is that it also quickly decreases* back down when it is not contracting (76). 

It is also important to remember that the high rates of carbohydrate oxidation during periods of physical activity come to an end when the activity bout ends (77, 78). We are unaware of any convincing human or animal findings that suggest otherwise. This is true even over a wide range of insulin concentrations during a euglycemic-hyperinsulinemic clamp (78). This is in part because, as stated above, the increase in energy demand by working muscle quickly plummets back down when stopping exercise. The half-time for the rise and fall in VO2 at the onset and end of contractile activity is only about 30 seconds (76). Also, even after some types of intense exercise when there is a small excess post-exercise oxygen consumption, it is dwarfed by the energy demand of even light activity (79). However, when this occurs the respiratory quotient indicates there is concurrently less total glucose oxidation than normal (77, 78). The net effect is that the total glucose oxidation during a postprandial period is reduced by previous exercise, and this can last for at least 7 hours (5, 6).

As discussed more later (see also **Supplementary Table** for annotated references), we hypothesize that the effectiveness of interrupting prolonged sedentary time (sitting inactive) by taking brief hourly activity breaks on glucose tolerance is limited *in part* because of the inability to stimulate glucose oxidation for a long enough duration relative to the more prolonged intervening periods of low metabolic rate during inactivity. Keeping this perspective in mind about the duration of muscular activity vs the amount of sedentary time helps to understand why we have proposed and tested the hypothesis (49) that specific methods of sustaining oxidative metabolism are needed.

### II. Glucose tolerance generally does not improve acutely in the minutes to hours after exercise bouts, in part because of opposing systemic influences

Even when insulin sensitivity is increased within the previously recruited muscles (1, 7, 8), there are generally other opposing systemic biochemical processes that may explain in part the difficulty to improve glucose tolerance during or following many kinds of acute bouts of activity (1–8). Unfortunately, the mechanisms that we discuss below are not discussed widely enough to penetrate deeply into the relevant literature. Even lengthy recent review articles in the most authoritative physiology journals have not addressed this much, or at all. *This has led people to frequently conflate an understanding that insulin sensitivity of the working muscle can increase after activity with the naïve misperception that this is also generally reflected in a robust improvement in postprandial glucose tolerance.* The integrative metabolism is likely much more complex. Cherrington’s group has even explained that not only is the fractional contribution of skeletal muscle to total body glucose uptake much less when glucose is ingested than during intravenous infusion, but also that there appear to be mechanisms that suppress skeletal muscle glucose uptake (at rest) to ensure appropriate partitioning of a glucose load (80).

#### Increased rate of appearance of the ingested glucose load

A significantly large rise in the rate of appearance of glucose into the blood during and after acute bouts of exercise can oppose other processes that would otherwise tend to improve glucose tolerance (1–4). One particularly insightful study by Knudsen et al. (4) should be widely read and discussed more often, both by basic scientists and clinicians. They found that glucose tolerance was not improved after acute exercise when testing subjects across the whole glucose tolerance continuum (T2D, IGT, and normal glucose tolerant controls). After completing an hour of moderate exercise at 50% peak aerobic power (~4.5 METs in the groups with prediabetes and diabetes), there was a higher uptake of glucose out of the blood during the first 90 min of the 3 hour postprandial test. However, there was not an improved postprandial blood *glucose concentration* compared to the inactive control trial in the subjects with T2D and IGT. The volunteers with normal glucose tolerance displayed significantly worse glucose tolerance after the moderate exercise (4).

This failure to improve glucose tolerance after exercise is not rare in the literature, e.g (1–8, 29, 39, 44, 50, 51). Beyond the inability to sustain a meaningful rate of carbohydrate oxidation after stopping a bout of contractile activity (discussed in the section above about muscle energetics), one of several other contributing factors is faster rate of appearance of the ingested glucose into the bloodstream after exercise (4). This is consistent with the evidence from responses in humans (2–4). It is also consistent with the findings by Hamilton and colleagues that measured rates of intestinal absorption of glucose more directly by surgical catheterization in dogs following treadmill exercise (1).

Blood glucose concentration in the postprandial period is a function of the rate of appearance of the glucose ingested. It should be noted that Knudsen et al. (4) and Rose et al. (2) reported that the rate of appearance of endogenous (hepatic) blood glucose was also significantly elevated during moderate exercise (4) and at rest (2, 4) during the early part of the postprandial period. Therefore, blood glucose concentration during and after bouts of acute physical activity is influenced both by potential for increases in the rate of intestinal absorption as well as the glucose uptake/release by the liver, and changes in the uptake by individual peripheral tissues. The exact dose-response for glucose tolerance and each of these factors during and after acute bouts of physical activity is largely unknown. However, it is reasonable to suspect that a homeostatic disturbance to a process like the faster intestinal absorption is somehow dependent on the magnitude that total body energy expenditure increases.

#### Peripheral insulin resistance beyond the previously working muscle fibers

Two independent studies provide evidence of a systemic process that causes an interesting type of acute insulin resistance in the hours after exercise (7, 8). Both studies used the arteriovenous limb balance method and isotopic tracers in combination with the euglycemic-hyperinsulinemic clamp technique. In one study, healthy men performed cycle ergometer exercise with the legs for 75 min. This caused insulin resistance in the non-working arm muscles during the post-exercise recovery period (8). This effect was strong enough to prevent any measurable glucose extraction by the arm muscles in either the fasted state or during insulin-stimulated conditions. Richter’s lab (7) extended this to find that local contractile activity by the quadricep muscle group in a single limb caused insulin resistance in the contralateral leg for at least 5–6 hours after the activity ended (~37% decrease in the insulin stimulated glucose uptake). The net systemic effect was to attenuate whole body insulin stimulated glucose uptake (7). Both this acute insulin resistance process and the elevated intestinal absorption of glucose described above would each (individually) tend to maintain higher levels of blood glucose concentration, and therefore oppose improved glucose tolerance. A teleological explanation is that if this was continued long enough into the post-contraction resting period, higher blood glucose levels might enhance glycogen concentration in muscles that had previously been glycogen depleted.

#### The lowering of glucose oxidation after ending bouts of acute activity

As described in the first section above, the acute increase in blood glucose oxidation during muscular exercise comes back down to normally low levels during muscular inactivity (77, 78), even when hyperinsulinemia is set to the same high level in both conditions (post-exercise and sedentary control) by an insulin infusion (78). Large muscle mass exercises like cycle ergometry can reduce postprandial glucose oxidation significantly for at least 5-7 hours in the recovery period, compared to remaining inactivity the same amount of time (5, 6). Therefore, our perspective is that the most effective way to sustain the benefits of oxidative metabolism by muscle mitochondria for long periods of time in the postprandial period is to sustain contractile activity throughout the entire period of hyperglycemia (**Figure 1B**).

#### Systemic factors that potentially limit the amount of glucose uptake during acute large muscle mass exercise

People obviously spend much more time sitting inactive than in the shorter amount of time within acute bouts of physical activity. Thus the above discussion of this section focuses first and foremost on what happens in the hours after ending a bout of activity. However, we need to be clear about two related issues to help sharpen perspectives of basic scientists and clinicians interested in developing how to better optimize muscular activity for improving glucose regulation. First, there is no doubt that contracting muscle can take up blood glucose to assist in fueling the *immediate* energy demands within the working muscle mass (81). Secondly, there may also be counteractive systemic processes to this immediate response during some types of physical activity (82, 83). As far as we know, Richter’s group (82) was the first to raise the hypothesis that the local rate of glucose uptake within a relatively small working mass (as determined by arteriovenous glucose uptake within a single limb during isolated leg extensions) is greatest when the muscle group is working in isolation at a relatively low total body energy demand (~24% of VO2max). Adding concurrent arm exercise to increase the total working muscle mass (yet still at a submaximal modest intensity of ~53% VO2max) caused a significant decrease in the leg glucose uptake (82). At least one study provided compelling evidence that blocking the normal beta-adrenergic receptor catecholamine stimulation at 45% VO2max increased blood glucose uptake 66% above normal (83). One of the potential reasons for this is because of a catecholamine-induced glycogen degradation in contracting slow-oxidative muscle fibers (84), which may normally restrain blood glucose uptake during physical activity that involves a large muscle mass (82). Below, we will return to these kinds of integrative insights when discussing the role of muscle glycogen vs blood glucose (section IV) and also later when describing the rationale for developing a small muscle mass activity focused on the soleus to improve metabolic health.

### III. The intrinsic phenotype of a muscle is critical to the biochemical responses to acute contractile activity

If a muscle lacks sufficient intrinsic molecular machinery to use blood glucose at high rates for oxidative metabolism, then logically the rate of glucose oxidation will be limited regardless of the total rate of ATP production. Conversely, a “high oxidative” muscle like the soleus has been shown to possess phenotypic qualities that influence the vascular delivery, tissue uptake, and cellular utilization of blood glucose and/or lipids (64, 71, 84–90). For example, the soleus metabolized about 6 times more blood glucose compared to other muscles like the adjacent fast-twitch glycolytic gastrocnemius muscle (88) during light ambulatory activity when hyperinsulinemia was set to a physiologically high value (~100 µU/ml).

Some of the many known intrinsic differences favoring health-enhancing responses in the soleus muscle include vascular differences favoring more delivery of blood-borne fuels and oxygen (85), up to 10 times more capillary bound lipoprotein lipase (90), higher hexokinase II and GLUT4 proteins regulating blood glucose (71), and less enzymes regulating glycogen utilization during contractile activity (86, 91, 92). Important to this, we and others suspect that the soleus (and maybe slow oxidative muscle fibers within other muscles to a lesser degree) is capable of rapidly switching between fueling the contractions with either blood glucose in the acute postprandial state or more lipids (including atherogenic plasma triglycerides) in the postabsorptive or fasted state (49, 89, 90). This “metabolic flexibility” could be very important for glucose tolerance, if the contracting muscle was capable of quickly increasing carbohydrate oxidation by a meaningful amount without most of that coming from glycogenolysis (discussed more below).

### IV. Muscle glycogen is generally the dominant carbohydrate oxidized to fuel the working muscles, not blood glucose

*Logically, for any given total energy demand by a working muscle there is a competition between substrates.* Thus, the amount of glycogenolysis in muscle is central to understanding fuel partitioning. This is because muscle glycogen is generally the dominant carbohydrate oxidized to fuel the working muscles, not blood glucose (60–63, 93).

Our perspective is that a high reliance on muscle glycogen for raising the carbohydrate ostensibly creates a type of competition with other substrates during contractions, including glucose oxidation. This is especially the case during the early minutes of activity, because blood glucose uptake increases slowly for up to about an hour after initiating physical activity (60, 62, 63), often without significant blood glucose uptake in the lower limbs during brief amounts of some types of activity (60, 62, 63, 94).

As shown in **Figure 1C** and discussed in more detail later, the soleus muscle definitely *has the potential* to work at a relatively intense local rate of oxidative metabolism with negligible reduction in the local soleus glycogen concentration while also having a large impact on postprandial glucose regulation (49). This was demonstrated in a study that compared prolonged inactive sitting to a type of contractile activity that targeted the human soleus while sitting comfortably for hours (**Figure 1C**). As described more in a later section below, this mode of targeted soleus activity (49), the soleus push up (SPU), is a specialized type of dynamic plantarflexion that raises the local energy demand a large amount despite working against a relatively low load. This is because it involves active shortening at a relatively high range of motion and velocity.

It is very important to emphasize that it would be incorrect of us or others to try to rush to generalize the negligible glycogen depletion we measured in the human soleus during prolonged SPU contractions (**Figure 1C**) to various kinds of “low intensity” activity, to all Type I fibers in other muscles used during other types of physical activity, or even to other types of soleus contractions. Convincing biopsy data by Jensen and Richter’s study (71) shows a rapid rate of glycogen depletion in the human soleus during a 45 min walk (**Figure 1C**). Furthermore, a classic original glycogen depletion study by Gollnick and Saltin (93) most clearly demonstrated that prolonged low intensity cycle ergometry exercise in healthy young men (~30% VO2max) causes profound glycogen depletion in Type I fibers obtained from vastus lateralis biopsies (a quadricep muscle). Finally, as shown (**Figure 1C**), our finding should also not be interpreted as evidence that all kinds of locally intense small muscle mass activity cause minimal changes in the intramuscular glycogen, such as in the vastus lateralis during isolated quadricep activity (61, 62).

The amount of glycogen reduction by a working muscle like the soleus during SPU contractions is likely influenced by the integrative whole-body cardiovascular, hormonal, and metabolic responses during the specific type of contractile activity. If so, this likely explains in part why rates of soleus glycogen lowering are less during SPU contractions than a large muscle mass activity like uphill walking (**Figure 1C**). However, the rate of muscle glycogen depletion is also influenced by the inherent local phenotype of each muscle. For example, glycolytic enzymes and glycogen phosphorylase are among the multiple proteins regulating different amounts of glycogen utilization in the soleus and other muscles (86, 91, 92). We found (49) that even untrained individuals could maintain a relatively intense local energy demand (i.e. high mass specific VO2 per kg by the recruited muscle, **Figure 1A**) when combined with only ~4% of the total activity energy expenditure accounted for by the lowering of soleus muscle glycogen (**Figure 1C**). It was feasible for these subjects to sustain the relatively low systemic energy demand with barely any increase in heart rate (49). *Given this, the SPU mode of contractions provides our lab and others in the field a method to better understand the capacity for the human soleus, under optimal conditions while there is negligible fatigue, to sustain for many hours a high oxidative metabolism (local mass specific VO2).*

Exercise physiologists have historically found it insightful to study the upper limits of fatigue resistance during prolonged contractile activity as a function of muscle glycogen utilization (70). The classic study about rates of glycogen utilization during exercise as a function of the “muscle quality” (high oxidative phenotype) in well-trained endurance athletes came from a study by Coyle’s group (70). They measured glycogen utilization during 30 minutes of cycling at 80% of VO2max (when total body VO2 was 3.8 L/min) in well-trained endurance cyclists with the same absolute aerobic capacity, but different skeletal muscle phenotypes. During the 30 min glycogen test in Coyle’s study (70), the calculated mass specific VO2 was ~230 mL/min/kg (assuming a non-muscle VO2 of ~0.3 L/min, and the 3.8 L/min VO2 was contained within a mass of ~15 kg muscle). This high mass specific VO2 is similar to the ~237 mL/min/kg muscle that we calculated for SPU contractions (**Figure 1C**), while at a very much lower total energy expenditure (49) compared to 80% of VO2max cycling. Importantly, the rate of vastus lateralis glycogen depletion in the athletes was also far greater than the changes in the soleus of our unfit volunteers. A remarkably well-trained/genetically endowed cyclist in that study (very high lactate threshold, percentage Type I fibers, capillaries per fiber, fatigue resistance during a performance test, and years of training) used less than one-half the glycogen of the other athletes when exercising at the same absolute and percentage of VO2max. Most interestingly, his vastus lateralis muscle glycogen reduction (~20 mmol/kg) in 30 minutes of exercise was similar to the soleus depletion of unfit people after SPUs in 270 minutes contractile activity at the same mass specific VO2 of the working musculature (49).

In summary, we believe understanding the substrate partitioning between glycogen utilization and other potential fuels is important for determining blood glucose concentration. Although the phenotypic qualities of skeletal muscle can be improved by consistent endurance training to reduce dependence on muscle glycogen, the human soleus apparently is capable of performing relatively *intense local contractile* activity without relying heavily on muscle glycogen. Taken together, the soleus is a good example of a muscle with the phenotypic qualities required to more rapidly induce the uptake of more blood-borne fuels (glucose after ingesting a glucose load, and lipids in the postabsorptive/fasted state).

## Interrupting prolonged sitting with brief activity breaks or standing

*We provide an annotated bibliography* in a **Supplementary Table**. This was compiled as a resource to evaluate the effects of at least 75 acute activity trials aiming to improve blood glucose in the postprandial period. Studies frequently tested *brief bouts* of walking (or other kinds of activity) that were of low duration (2–6 min bouts). The cumulative hourly duration was also often for a low duration, most commonly ~4–6 min/hour (*e.g. repeating a 3 min walking bout 2X per hour for a total of ~30 minutes of physical activity over a 5 hour postprandial period*). This table is focused on including studies specifically taking aim at solving problems resulting from prolonged sedentary time caused by sitting, not on traditional exercise prescriptions (e.g. a single bout of continuous exercise). The exception to this is a subset of studies that highlighted misunderstood/overlooked transient physiological process that impact postprandial glucose regulation in the hours following a bout of exercise, as already discussed in the earlier sections of this article.

Studies representing a wide range of intensities and modalities of activity were evaluated. This includes standing still (sometimes for the entire postprandial period), slow-moderate walking, vigorous treadmill exercise, and even calisthenics/resistance exercises to activate a large muscle mass similar to circuit training.

It is helpful to put the intensity, duration, and caloric volume of these studies in context of the U.S. physical activity guidelines (95). There is no stated intensity or duration in the guidelines for reductions in total “sedentary time” (i.e., how to deal with the large amount of time people spend sitting inactive). However, when it comes to “physical activity”, the most widely understood physical activity recommendation is for all able-bodied people to expend the equivalent weekly energy expenditure of 150 minutes at 3 METs (e.g. 75 minutes at 6 METs would be equivalent volume). There are no longer any requirements for the activity frequency in those guidelines. In short, someone could accomplish this by walking at 3 METs for 25 minutes on 6 days of the week. Although most of these studies in the **Supplementary Table** were explicitly aiming to test for the effects of “sedentary behavior interruption” with small amounts of light activity, many unintentionally ended up with a protocol that was enough volume to be in the range of the traditional “physical activity” recommendations. For example, studies in this field that used slow walking generally included a speed of ~2 mph often for a duration up to ~6 min or more per hour for 4 or more hours, which is a metabolic rate close to ~300% of resting metabolic rate (~3 METs). Our opinion is that these durations of contractions actually are insufficient to overcome the *distinct* metabolic problems caused by *far more* daily sitting time.; i.e., living most of the day with insufficient muscle metabolism to stimulate processes like glucose oxidation.

The standing studies used relatively longer durations of about 30 min/hour (17, 19, 20, 22) and sometimes continuous standing for 2 or more hours (10, 13). Those studies resulted in either nonsignificant or small effects regardless of the standing time. One study was particularly interesting and appears to be carefully executed because they used an OGTT, while EMG was worn on multiple thigh muscles, and VO2 and RER were measured (13). That study reported a 9% increase in VO2, with a small but significant *decrease* in carbohydrate oxidation, and a statistically significant *worsening* in the glucose tolerance response during standing compared to sitting (13). Recently a study (9) found that 10 min standing breaks did not improve glucose tolerance significantly in participants with prediabetes. Finally, one of these standing studies (15) found that it did not matter whether the postprandial test was with a 75 g OGTT or a mixed meal containing a carbohydrate load.

In general, most of the brief breaks studies did not reduce blood glucose concentrations at all or by much magnitude, regardless of the specific standing and walking patterns. One of the most comprehensive studies evaluated 3 levels of either 2, 6, or 12 min of walking per bout without a significant effect of any of these durations (34). From these kinds of walking studies, there is no consistent evidence for an obviously superior brief break pattern. Similarly, the magnitude of glucose change during the postprandial period in the many different low to moderate intensity walking trials had a median reduction of about -5 mg/dL (10, 11, 14–16, 18, 21, 23–36). This was similar to the effect in studies (10, 37–40) testing much more vigorous intensity treadmill and cycling breaks (median glucose difference of -3 mg/dL). Also, circuit training type of brief calisthenic exercises aimed at engaging a large muscle mass (24, 27, 30, 35, 41, 43) did not consistently cause a convincing difference from prolonged inactive sitting near resting metabolic rate (median glucose difference of -4 mg/dL).

Comprehensive reviews point out the need for more *chronic* sedentary behavior intervention studies that clearly and completely quantify the total sitting time and the patterns of physical activity throughout the day when testing for glucose tolerance and lipid responses (96). In our opinion, the most thorough and well described test of frequent activity breaks from sitting (FABS) was in a rare multiweek intervention (97). The finding that the FABS intervention did not improve glucose tolerance was clearly demonstrated. The activity breaks did reduce fasting blood glucose levels by a small amount and also the daily glucose variation compared with baseline. The logical interpretation by the authors was that FABS approach did not induce large enough volumes of activity to contend with the large amounts of sedentary time in the modern lifestyle. Although it is beyond the scope of this perspective to discuss in detail the intervention studies lasting weeks to months, the metabolic responses reported thus far are either non-existent or very small (96). These findings illustrate the pressing need to improve the methods for reducing/interrupting sedentary time more than has been studied to date.

## What is the time course of contraction-mediated glucose uptake and oxidation to fuel a bout of acute physical activity?

Given the difficulty in reducing blood glucose by brief bouts of muscular activity, one important insight that has been overlooked deals with studies that evaluated the time course for glucose uptake by skeletal muscle. This has obvious relevance to understanding the limitations of muscle to take up and use blood glucose as an immediate energy source at the onset of activity, especially during brief bouts of physical activity. Studies found that muscle glucose uptake appears to sometimes have a relatively slow and gradual time course at the onset (in the first 30 minutes) of continuous contractile activity (60, 62, 63, 94). In studying the time course in the human lower limb musculature during cycling, there was no increase in glucose uptake in the lower body musculature after either 5 or 15 min of continuous contractions, and significant responses were not evident until after 30 min (63). Furthermore, others also showed a slow response for glucose uptake during extensions of the isolated quadriceps muscle (62). One study was designed specifically to study the delay in glucose uptake by using a sophisticated tracer technique in the isolated contracting hindlimb of rabbits. The study found that the increase in rate of blood glucose uptake was delayed for over 5 minutes after the onset of contractions (94), consistent with the aforementioned human studies finding that the initial increase in carbohydrate oxidation is caused by a more rapid increase in glycogenolysis (62, 63).

There is no doubt that exercise of sufficient duration in the postprandial period has the potential to decrease the glucose concentration at some time points. However, careful inspection of the time course is important because the postprandial period for glucose excursions can take up to 3 hours or more. *Importantly, studies often demonstrate that when even sustained physical activity (e.g. treadmill or cycling exercise) ends within the postprandial period and glucose is still rising or near the peak, once the exercise stops the glucose concentration can rapidly rebound back up to the sedentary control levels, or even significantly above control levels at some time points* (29, 39, 98). For this reason the average glucose over the entire postprandial time period (e.g. total postprandial glucose rise above fasting, or iAUC) may often not be improved compared to sedentary control levels, even when moderate or vigorous physical activity causes a transient decrease in glucose concentration within the first part of the postprandial period (29, 39, 51, 98). However, this is not surprising given what was described above about VO2 and glucose oxidation returning to low levels after stopping muscle contractions (77, 78), combined with the processes we reviewed above that tend to oppose glucose lowering after stopping a bout of activity despite potentially increased insulin sensitivity in the recruited muscle mass.

Effective resistance exercise aims to induce an even more intense muscular recruitment of multiple muscle groups for brief periods of contractions than during aerobic endurance exercise. Although it is beyond the scope of our purpose here for a thorough review of many resistance exercise studies, the **Supplementary Table** does provide some citations that specifically focused on using simple resistance exercise (see “Calisthenics Studies”) to frequently interrupt sedentary time (prolonged inactive sitting). There may be some important similarities with regards to the transient nature of glucose regulation between an intense circuit of resistance exercises and traditional aerobic endurance exercise. For example, Kanaley’s group reported that when resistance exercise (99) or aerobic treadmill exercise at 65% VO2max (39) was timed to coincide with the early postprandial period, there appeared to be a temporary reduction in glucose concentration that quickly rebounded back up at least to the level of a sedentary control trial soon after both types of acute exercise ended.

**Figure 2:**
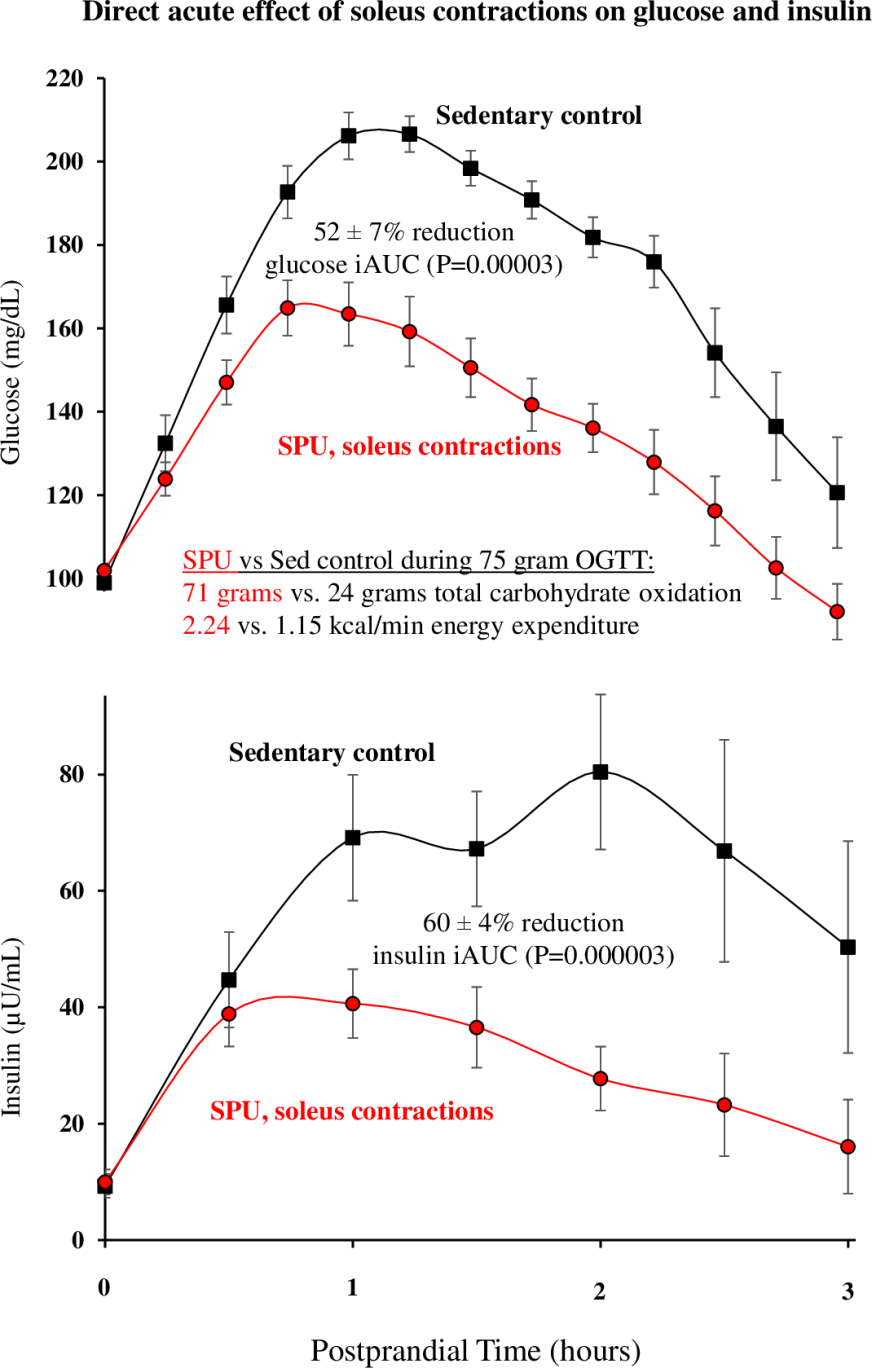
The postprandial glucose and insulin responses to acute contractions targeting the soleus muscle. The blunted glucose excursion began in the early postprandial phase when glucose was still rising (e.g. statistically significant effects of SPU contractions by 30 min) with a sustained effect during the continuous contractile activity. This was from a randomized crossover experimental design (subjects serving as their own control) when the muscular activity increased the total energy expenditure by ~100% above sitting inactive in the control trial (P=0.000004). During the 3 hour OGTT, there was a sustained increase in the total carbohydrate oxidation (P=0.000004), enough to almost triple the total body carbohydrate oxidation (71 ± 5 vs. 24 ± 3 grams, P=0.000004) to a level almost equal to the 75 gram oral glucose load. For clarity in this figure, only one of two levels of SPU trials tested is shown here, but both demonstrated a statistically significant effect on the same outcomes. There was a dose-response (not shown) between the intensities for glucose (P=0.047), insulin (P=0.001), and an index for an integrated postprandial metabolic response (P=0.007). Figure adapted from (49).

Because resistance exercise training is often aimed at increasing total body skeletal muscle mass, a common misperception is that it is important to add more muscle mass in order to create a larger “metabolic sink” for taking up substrates like blood glucose and lipids. However, this is a mistaken notion for why muscular activity is important for metabolism. First, any straightforward quantitative calculation reveals that it is impossible to add enough muscle mass to cause a quantitatively meaningful change in either blood glucose storage or oxidation in resting muscle. For example, recall that we explained above that at rest, the entire skeletal muscle mass accounts for only about 15% of the whole-body glucose oxidation (69) and ~30% of the total blood glucose disposal (oxidative and non-oxidative utilization) in the postprandial state (69, 80). Therefore, the direct effect of even a 50-100% increase in whole-body muscle mass (an impossible expectation of resistance training) would by itself not have much effect compared to acute contractile activity. In contrast to this and described in the next section, recent research has shown that a muscle mass as small as ~1% of body weight (the soleus muscle) is sufficient to raise whole body glucose oxidation to about 300% of sedentary control levels. This is more than the *entire resting* muscle mass and all other organs combined (**Figure 1B**) and a sufficient amount of working muscle mass to by itself cause a large increase in glucose tolerance (**Figure 2**).

In summary, the above findings provided us a rationale for the need to develop a method to test the hypothesis that glucose tolerance could be improved by a large magnitude, if that approach could sustain a meaningful increase in muscle VO2 throughout the entire postprandial time period (49).

## The goal to develop a type of muscular activity specifically geared to improve metabolic health

With the above principles in mind, our recent study (49) began to describe a unique approach to induce and sustain an exceptionally high rate of local oxygen consumption while targeting activation of the soleus (**Figure 1A**). It was important to first test how to raise the rate of soleus oxidative metabolism as much as possible for prolonged periods in unfit people (49). This was accomplished with a specific type of plantarflexion that focuses on instructing volunteers how to induce a relatively high soleus EMG (electromyography, a measure of muscle activation) during a moderately high range of motion/velocity motion. It is particularly important that the soleus activation (EMG on time) is within the “concentric” phase of plantarflexion (i.e. when the soleus muscle is shortening), which we showed is unlike the more isometric/slightly eccentric type of soleus contractions that take place during walking (49). This can be executed in normal chairs when the ankle and knee joints are positioned adequately. There is not a need to add external resistance because the mass of the lower limbs is a sufficient load when the movement is executed correctly. We simply termed this very specific kind of plantarflexion movement a soleus push up (SPU). Supervision was provided to ensure the participant was able to sit comfortably over long periods of time in a relaxed position in both the SPU and sedentary control trials.

Our hypothesis was that a small muscle mass, working at a relatively very low total body energy expenditure (and low total carbohydrate oxidation), may not only be sufficient to improve glucose tolerance, but even well geared for the task if the several related conditions are met, as already discussed and referenced above. Briefly, those factors include the following, 1) a high duration of contractile activity that can be sustained feasibly throughout the postprandial period (49); 2) a limited contribution of glycogen to supply the carbohydrate oxidation during contractions (49); 3) a focus on the specialized phenotype of the high oxidative soleus because in all animals it has been studied, and humans alike, it is enriched with the molecular machinery with regulatory proteins (71) favoring high rates of blood glucose uptake and utilization in some conditions; 4) and the potential for enhancing processes favoring local glucose utilization by a small recruited muscle mass when there is a low total energy demand/intensity of effort, thereby attenuating counteractive systemic homeostatic disturbances (82).

Importantly, the generation of this hypothesis was also informed by a simple quantitative theoretical model that describes the magnitude that glucose oxidation would need to increase to cause a large clinically significant improvement in glucose tolerance (49). This mass balance model predicted from the extracellular glucose distribution volume and blood glucose concentrations, that in theory carbohydrate oxidation only needs to increase by 100–200 mg/min (0.4-0.8 kcal/min muscular energy) above the normally low resting levels in the postprandial period to cause a large and rapid acute effect on glucose tolerance. One condition of this model is that muscle glycogen is not the predominant carbohydrate for the oxidative metabolism. We estimated that the soleus muscle alone could be sufficient to sustain 100–200 mg/min of glucose oxidation during SPU contractions. Therefore, we performed a dose-response test of two levels in a repeated measures design to determine if this kind of targeted low-effort muscular activity causes meaningfully large improvements in glucose tolerance (49).

During one of the 3 hour OGTT trials (**Figure 2**), we tested SPU contractions when there was a modest increase in the total body energy demand of ~1.7 METs. The average intensity in this trial was equivalent to ~100% increase in the total body energy expenditure of sitting at rest. Using a randomized cross-over design, this level of non-fatiguing activity resulted in 52% less rise in postprandial glucose concentration despite 60% less hyperinsulinemia compared to the same subjects sitting inactive in a sedentary control trial on another day (**Figure 2**). A dose-response comparison revealed that a lower SPU contractile intensity at half the above level still produced significant, albeit smaller changes (i.e., ~40% reductions in both the glucose and insulin excursions during the OGTT). The remarkable improvements in the glucose tolerance were statistically significant no matter how we subdivided these participants (by age, gender, BMI, habitual daily sitting time, habitual steps/day, and fasting glucose) (49). Examination of the time course (**Figure 2**) revealed that this kind of muscle contraction was already effective quickly within the first phase of the OGTT when the glucose was still rising. Thereafter the glucose, insulin, and C-peptide concentrations remain lower than the sedentary control levels (49).

Importantly, this singular movement targeting the soleus muscle demonstrated fatigue resistance in relatively unfit volunteers (VO2max of 30 ml/kg/min), likely in large part because this method utilized minimal glycogen despite an intense *local* energy demand (**Figure 1C**). We are completing follow up studies to better understand why glycogen is not used and to directly quantify the rates of glucose utilization. The negligible glycogen response during SPU contractions (**Figure 1C**) may have benefitted from the intrinsic phenotype favoring high blood glucose metabolism (71, 86–88, 91, 92), especially when the local contractile activity is not part of a large muscle mass activity (82) and when the systemic homeostatic responses stimulating glycogenolysis are negligible (84). We confirmed that by restricting the work to a small muscle mass with a relatively subtle rise in total body energy expenditure, there were limited homeostatic disturbances involving the sympathetic nervous system, such as almost no change in heart rate and blood pressure (49).

The SPU activity was sufficient to maintain the calculated total blood glucose oxidation at ~253 mg/min above the inactive control level (49). To put this rate in perspective, the larger muscle mass involved in cycle ergometry at a moderate intensity (5 METs) raised the total carbohydrate oxidation much more to ~1600 mg/min (63). However, once the 10–15 times greater rate of glycogen breakdown during cycling vs SPU activity is accounted for (49), the calculated oxidation due to blood glucose in the entirety of both legs during cycling was just ~180 mg/min (63), which was not greater than what could be sustained indefinitely by the much smaller soleus muscle during SPU activity.

As discussed above, a smaller than normal competition between glycogen and glucose (**Figure 1C**) may be part of the explanation for the potency of this single muscle group with a relatively small increase in total body expenditure (2.24 during SPU contractions vs 1.15 kcal/min during the sedentary control condition during the OGTT) to cause a large reduction in the glucose excursion despite much less hyperinsulinemia (**Figure 2**).

We also reported in that study the potential impact of SPU contractions on total fat oxidation while reducing the VLDL-triglyceride concentration during the transition to fasting between meals (49). Our current studies are developing a more clear understanding of how a small muscle mass activity like this can in some ways create even more meaningful systemic changes in the metabolism of distinct classes of lipids than for blood glucose regulation. As far as we know, this is the first example of physiological research being used to develop a type of contractile activity specifically geared for a high duration of locally intense (but non-fatiguing, low effort) slow oxidative muscle metabolism. Interestingly, a repeated measures study was recently published that supported our findings of the potency of soleus push up contractions on oral glucose tolerance (47). In this study, participants with prediabetes had a significant lowering of hyperglycemia during a 2 hour OGTT. Two trials with SPUs were reported, but in neither was the actual metabolic intensity and carbohydrate oxidation measured. Although the ROM and VO2 was not monitored to optimize the SPU movement, they did demonstrate that the average glucose throughout the 2 hour glucose tolerance test was reduced by -32 mg/dL when soleus EMG feedback of the SPUs was provided, and by -24 mg/dL without their EMG biofeedback (47).

## Discussion

It is safe to expect that there is widespread agreement that living with inactive muscles a large amount of time each day in the modern world is not good for human health. The crux of the issue is what to do about it.

It is helpful to realize that large volumes of sitting is ubiquitous in the modern world; >95% of adults of all ages sit >50 hours/week (100, 101). A whole room calorimeter study of ours concluded that the time people spend sitting is not meaningfully different from resting metabolic rate or even during periods of bedrest (74).

We believe that solving the *distinct* problems caused by the sluggish oxidative muscle metabolism during large amounts of sedentary time is best solved by directly replacing large amounts of that time with low stress muscular activity (e.g. SPU contractions). One perspective herein is that depending on the type of physical activity, attempts to raise the whole body intensity may be counterproductive when it leads to a lower duration and invokes physiological processes that tend to oppose a glucose lowering after ending the exercise bout. The physiological principles summarized within this article are intended to hopefully provide a physiological framework to guide future research.

There is no debate that understanding skeletal muscle metabolism is important for glucose tolerance and many aspects related to chronic disease prevention and healthy aging. A mechanistic understanding of the integrative biochemistry involved helps point the way to the problems and potential solutions. For example, despite being the largest lean body tissue, the entire skeletal muscle mass contributes only ~15% to the oxidative metabolism of glucose following carbohydrate ingestion when resting (69), as calculated from the best data available (arteriovenous glucose oxidation measured in the lower limb of middle age overweight individuals). In our perspective, this points the way toward the need to understand the effects of low stress contractions (subtle, but energetically meaningful) that can be sustained by a high quality slow oxidative muscle for large amounts of time each day (for hours, not minutes).

Otherwise, there is in our opinion a greatly untapped potential for high oxidative muscle to make a more impactful contribution to processes like blood glucose oxidation. In short, from a substrate oxidation perspective, it does not matter how much muscle mass is in the body or how much mitochondria muscle contains if there is not a stimulus for sustaining a meaningful rate of oxidative metabolism. This is useful in developing innovative solutions to capitalize upon the molecular machinery in muscle. Amplifying distinct processes like increased oxidative muscle metabolism was found to provide a potent improvement in postprandial glucose tolerance and greatly reduce hyperinsulinemia (49). Paradoxically, the common excuse of needing to “make time” to interrupt prolonged sitting to sit less and exercise more does not apply well to this particular type of prolonged local contractile activity by the soleus. SPU contractions are by design done during ordinary sitting, which people do a large amount of time each day anyway (100, 101). Fortunately, this method of raising soleus contractile activity is possible during sustained fatigue-resistant voluntary contractile activity in unfit individuals who are at risk for diseases associated with impaired glucose tolerance (49). The current article is limited to only discussing the acute responses (direct effects of contractile activity). The current article is also limited to focusing only on the potential direct effects of raising oxidative metabolism of blood glucose. Thus, we believe it will be a fascinating and important scientific advancement to eventually describe multiple other adaptive responses caused by replacing >20–40 hours of weekly sedentary time with an equally large amount of local soleus muscle contractile activity.
