# Supplementary material for: Physiological processes induced by different types of physical activity that either oppose or enhance postprandial glucose tolerance
Source: Front Endocrinol (Lausanne). 2025 Jul 4;16:1601474. doi: 10.3389/fendo.2025.1601474 (PMC12272061; doi:10.3389/fendo.2025.1601474)
Supplement: Supplementary file 1 [file DataSheet1.pdf]

Supplementary Table. Annotated bibliography of 79 activity trials of 49 mostly ambulatory and other large muscle mass activity publications attempting to improve postprandial glucose concentration compared to sitting inactive.

**STANDING ACTIVITY STUDIES TO INTERRUPT SITTING SUMMARIZED**<br>The median reduction of postprandial glucose of these 16 standing trials from 15 publications was -3 mg/dL. Ten trials from 9 studies using standing as brief breaks from sitting (i.e. <50% of the time doing standing activity) showed a median change of -2 mg/dL. The four trials that used high duration of standing breaks (50% of sitting time replaced) had a median glucose change of -4 mg/dL. One of the two studies with continuous standing in the postprandial period had a mild-moderate but significantly worse glucose tolerance to the two hour OGTT, while the other had no significant effect after a longer time period and larger carbohydrate meal.			
**Study**	**Activity**	**glucose Δ mg/dL**	**Annotated Comments**
Toledo 2023 (9)	Standing for 10' each 60'	-2.7 NS	Interrupting sitting with 10 min standing breaks each hour did not impact the postprandial glucose excursion in people with prediabetes
Peddie 2021 (10)	300' *continuous* stand	-4 NS	The most prolonged standing duration of any of these studies. Designed to mimic a standing desk with computers, reading etc. No improvement in the 5 hour response to a 170 g carbohydrate meal.
Yates 2020 (11)	5' stand / 30'	0 NS	A relatively large study group was subdivided for comparisons and there was no improvement in glucose tolerance regardless of sex, age, and ethnicity.
Altenburg 2019 (12)	10' stand / 60'	+1 NS	Intermittent standing as 10 min breaks each hour did not alter postprandial glucose concentration.
Gao 2017 (13)	120' *continuous* stand	+13*	Glucose response was significantly elevated during standing vs sitting in a 2 hour OGTT. VO2 was measured with a 9% increase during standing. Carbohydrate oxidation was reduced significantly by standing. EMG of the large muscle mass in the anterior and posterior thigh increased significantly, but by a small amount.
Kerr 2017 (14)	2' stand /20'	-5 NS	Results of minor differences was in agreement with other studies
Pulsford 2017 (15)	2' stand/ 20'	-2.1 NS	Glucose responses during neither a standard OGTT nor a mixed meal were improved by frequent standing breaks every 20 min
Brocklebank 2017 (16)	2' stand/ 20'	-4*	iAUC was significant yet of a small magnitude of 4 mg/dL, but the AUC glucose was not significantly different with frequent standing breaks every 20 min
Benatti 2017 (17)	15' stand/30'	-4.7*	Longer breaks for 15 min each half hour produced a small but statistically significant reduction in the postprandial blood glucose response
Kerr 2017 (14)	10' stand / 60'	+2 NS	A good comparison of standing vs sitting and walking
Henson 2016 (18)	5' stand / 30'	-4*	A small magnitude reduction that was statistically significant in overweight or obese post-menopausal women with prediabetes
Hawari 2016 (19)	15' stand/30'	-3 NS	Two patterns of standing. The less frequent approach raised energy expenditure over 8 hours period by 11%. The more frequent standing bouts raised the energy expenditure by 21%, yet had no extra dose-response effect on postprandial glucose in obese men.
	Many more frequent 1.5' stand breaks	+1 NS	
Crespo 2016 (20)	Up to 30' of standing per 60'	-5*	Interrupting sitting with up to 30 min breaks per hour did not alter postprandial glucose concentration.
Bailey 2015 (21)	2' stand/ 20'	+0.7 NS	Interrupting sitting with frequent breaks each 20 min did not alter postprandial glucose concentration.
Thorp 2014 (22)	30' stand/ 60'	-3.6*	Interrupting sitting with 30 min breaks each hour significantly lowered postprandial glucose concentration by a small amount.
**INTERMITTENT BRIEF WALKING BREAKS AT A LIGHT TO MODERATE INTENSITY**<br>The median reduction of these 33 low moderate intensity walking break trials from 23 studies was -5 mg/dL.<br>Most of these were at 2 mph, which is about 3X RMR (resting metabolic rate) which is about 3 METs (moderate intensity).			
**Study**	**Activity**	**glucose Δ mg/dL**	**Annotated Comments**
Chueh 2025 (23)	3'walk/30'	-6*	A small magnitude that was statistically significant by interrupting sitting each 30 min to walk
Biddle 2025 (52)	5' upright activity/30'	-4*	Overweight or obese with prediabetes and diabetes (HbA1c: 5.7-7.5%). Authors concluded the glucose change was "not clinically meaningful". Upright activities chosen included walking, and combined with resistance exercise.
Gao 2024 (24)	3'walk/45'	-5*	A small magnitude of effect that was statistically significant.
Duran 2023 (25)	5'walk/60'	-4 NS	4 patterns of walking over an 8 hour test day with two postprandial tests in the same day. Only the 5 min walk break every 30 min significantly reduced postprandial glucose compared to a prolonged sitting control trial. As with Yates 2020 below, this is a high amount of extra daily steps
	5'walk/30'	-12*	
	1'walk/60'	-2 NS	

	1'walk/30'	-6 ^{NS}	to add (~8000 steps/8 hours), given that many prior pedometer lifestyle studies typically report increases of about only 1000-2000 steps/day.
Correia 2023 (26)	2'walk/30'	-2 ^{NS}	No effect of walking breaks on postprandial glucose in elderly participants when they were either detrained or trained.
	2'walk/30'	-6 ^{NS}	
Gillen 2021 (27)	2'walk/30'	-5 ^{NS}	This study had participants interrupt prolonged sitting every 30 min with 2 min of walking.
Peddie 2021 (10)	2'walk/30'	-3 ^{NS}	This study had participants interrupt prolonged sitting every 30 min with 2 min walking.
Pinto 2021 (28)	3'walk/30'	-5*	Study was performed in people with rheumatoid arthritis.
Wheeler 2020 (29)	30' walk<br>3'walk/30' + 30'walk	+2*<br>+3*	A small magnitude of statistically significant change, but in the direction of worse glucose tolerance after 2 patterns of walking breaks.
Yates 2020 (11)	5'walk/30'	-5*	Both this and Duran 2023 (above) showed that 5' walk breaks/30' over 7-8 hrs lowered glucose -5 to -12 mg/dL with a high amount of daily steps to add (adding ~8000 steps per each 8 hours)
Crespo 2016 (20)	Up to 30'/hr	-9*	This high amount duration of walking up to 30 min/hr (average ~20' walk/hr for the 6 hr AUC in the lab) was the most walking of any of the breaks studies and it was performed at a lower speed of 1 mph in overweight/obese participants.
Larsen 2015 (42)	2'walk/20' 1st day	-3*	In overweight and obese adults, 3 continuous days of breaks vs. prolonged sitting did not add any additional benefit to glucose lowering compared to 1 day.
	2'walk/20' 3rd day	-4*	
Maylor 2019 (31)	2'walk/30'	+4 ^{NS}	This study had participants interrupt prolonged sitting every 30 min with 2 min walking.
Dejong 2019 (32)	5'walk/60'	0 ^{NS}	This study had participants interrupt prolonged sitting every 60 min with 5 min walking.
Paing 2019 (33)	3'walk/30'	-10 ^{NS}	This study had participants interrupt prolonged sitting every 30 min with 3 min walking.
	3' walk/15'	-16*	This was more frequent walking than most studies (each 15 min walked for 3 min). The cumulative walking time was 12'/hr over 8 hours for 96' min total walking in the day (compared to prolonged sitting inactive), with a total of about 10,000 extra steps/8hrs. This is a high amount of daily steps to add compared to many free living pedometer intervention studies.
Thorsen 2019 (34)	12' walk/120'	+1 ^{NS}	This is the most comprehensive experimental study to study the effects of activity patterns independent of total sedentary time, while also making measurements of oxidative metabolism. Regardless of the pattern, the glucose tolerance was not improved by either interrupting sitting frequently (each 20 min) or by taking longer breaks when interrupting sitting once an hour.
	6' walk/60'	+2 ^{NS}	
	2'walk/20'	-2 ^{NS}	
Brocklebank 2017 (16)	2'walk/20'	-4 ^{NS}	This study had participants interrupt prolonged sitting every 20 min with walking for 2 min.
Kerr 2017 (14)	2'walk/60	-6 ^{NS}	A good comparison of walking vs standing and sitting.
Pulsford 2017 (15)	2'walk/20'	-6*	This study had participants interrupt prolonged sitting every 20 min with walking. The mechanism is unknown and like the other "break studies", VO2/carbohydrate oxidation was not measured.
Dempsey 2016 (35)	3'walk/30'	-24*	This study had participants interrupt prolonged sitting every 20 min with walking.
Henson 2016 (18)	2'walk/20'	-5*	The 2 minute walking breaks every 20 min was associated with 5 mg/dL lower postprandial glucose.
Bailey 2015 (21)	2'walk/20'	-13*	The 2 minute walking breaks every 20 min was associated with 13 mg/dL lower postprandial glucose.
Dunstan 2012 (36)	2'walk/20'	-6*	Prolonged sitting was frequently interrupted with 2 different intensities. There was no advantage to a more brisk walk than 2 mph (~3 METs), with both levels of walking intensity giving very similar glucose lowering responses. The higher intensity was relatively difficult for these volunteers because it had to be adjusted downward in some people in order to complete the protocol.
	2'walk/20'	-7*	

BRIEF ACTIVITY BREAKS AT A MORE VIGOROUS INTENSITY SUMMARIZED

These studies focused on testing exercise at a more vigorous intensity than most studies listed above. The median change in glucose caused by these activity breaks was -3 mg/dL.

Study	Activity	glucose Δ mg/dL	Annotated Comments
Engeroff 2022 (37)	6’ cycling/60’	-4 ^{NS}	70% VO2max cycling exercise performed for 6 min each hour
Peddie 2021 (10)	2’ activity/30’	-3 ^{NS}	3.1 mph /10% grade (~5.3 METs intensity from uphill walking)
Homer 2017 (38)	2’ activity/30’	-1 ^{NS}	60% VO2max treadmill exercise
	2’ activity/30’ + 30’ continuous activity	-2 ^{NS}	60% VO2max treadmill exercise
Holmstrup 2014 (39)	5’ activity/60’	-2 ^{NS}	There was no improvement in glucose tolerance in these obese young adults with IGT (impaired glucose tolerance) when taking brief breaks at a relatively high intensity of 60-65% VO2max treadmill exercise.
Peddie 2013 (40)	1.7’activity/30’	-5*	60% VO2max treadmill. The exact glucose concentrations were not reported, and thus we estimated this small magnitude of change during the postprandial period from their Figure.

CALISTHENICS STUDIES. THESE INVOLVED LARGE MUSCLE GROUP EXERCISES USING EITHER A CIRCUIT STYLE RESISTANCE TRAINING APPROACH OR VARIATIONS ON BODY WEIGHT SQUATS.

The median lowering of glucose by these activities was –4 mg/dL. This includes studies used squats. Other studies using “standups” or a circuit of “simple resistance activities” to increase the muscular activity of multiple large muscle groups (half squats, calf raises, gluteal contractions, and knee raises). Each of these involve compound muscular movements to engage multiple large muscle groups in the lower limbs and trunk. Acute uninterrupted sitting is the reference sedentary control condition. The duration was extended for up to 8 hours of treatment in some of these studies. The early study (Dempsey 2016) showed a -24 mg/dL lowering of postprandial glucose, but 5 years later, the exact same methods in the same type of patients (ie T2D) from the same lab showed no effect on postprandial glucose, nor 3 years later in inactive obese middle age people (Larsen 2019). In 2021, they showed that a less frequent pattern (6’ SRA break/60’) in T2D showed a significant -11 mg/dL lowering while the 3’ break/30’ did not.

Study	Activity	glucose Δ mg/dL	Annotated Comments
Homer 2021 (41)	3’ SRA/30’	-4 ^{NS}	This more recent study by Homer and colleagues (2021), tested the circuit resistance training approach (SRA) for engaging a large muscle mass. The study completed multiple circuits within each 3 or 6 min break, which was repeated every 30 min. The study in T2D patients found that the 3 min exercise bouts did not significantly lower postprandial glucose compared to sitting inactive, but the 6 min bouts did lower glucose. The lack of effect from the 3 minute resistance exercise bouts was in general agreement with the earlier finding by Larsen (2019), but inconsistent with an earlier study (Dempsey 2016)
	6’ SRA/60’	-11*	
Larsen 2019 (30)	3’ SRA/30’	-4 ^{NS}	There was not a significant glucose lowering in people who were obese when interrupting prolonged sitting to take 3 min calisthenic breaks every 30 min.
Dempsey 2016 (35)	3’ SRA/30’	-24*	The SRA activity was distributed in 3 min bouts each 30 min. See notes above in the more recent follow up study by Homer in 2021 about the inconsistency of these 2 studies testing patients with T2D.
Gao 2024 (24)	10 squats/45’	-5*	This study found a small but significant improvement in glucose tolerance with squatting exercises instead of prolonged sitting.
Gillen 2021 (27)	15 standups/30’	-2 ^{NS}	This study and the earlier one below both evaluated a practical variation to the calisthenic approach, by having people get up from sitting frequently in order to engage the trunk and lower limb muscles.
Hawari 2019 (43)	10 standups/20’	0 ^{NS}	This study found that standing up multiple times every 20 minutes did not change postprandial glucose concentration.

TRADITIONAL ENDURANCE EXERCISE: TESTING THE GLUCOSE TOLERANCE IN THE MINUTES TO HOURS AFTER COMPLETION OF WELL CONTROLLED BOUTS OF MVPA IN LABORATORY EXPERIMENTS

These studies tested glucose tolerance after a wide range of times after ending an acute bout of exercise. In general, they all demonstrate that postprandial glucose concentrations are either not changed or higher when the postprandial test began minutes to hours after finishing a bout of exercise. There was a wide range in the duration of the exercise bouts, lasting from 30 to 180 minutes.

Study	Activity	glucose Δ mg/dL	Annotated Comments
Flockhart 2023 (44)	EX for 3 hours 65% VO2max<br><br>EX for 3 hours 65% VO2max	Glucose tolerance sig worse after EX<br><br>Glucose tolerance not diff after EX	Endurance trained individuals tested 14 hours post exercise; glucose concentration was significantly 20-25 mg/dL higher during the 45-120’ period of the OGTT.<br>Untrained individuals exercising at the same relative intensity; no effect of the prolonged exercise on glucose tolerance 14 hours after exercise.
	HIIT intervals (5x4’ at 95% VO$_2$max)	No effect on glucose tolerance	High intensity intervals performed 14 hours before OGTT did not improve or worsen the glucose tolerance in either endurance trained nor untrained participants.
Parker 2021 (6)	1 hour at 70% VO2max	Glucose tolerance sig worse after EX	75 g OGTT began 3 hours after exercise ended. Almost the same response as the study above by Flockhart, there was a 20-25 mg/dL higher glucose after exercise during 45-120’ of OGTT than when the same individuals remained inactive in a control trial. Authors conclude ~40% decrease in postprandial glucose oxidation (~100 mg/min) impaired glycemia. When the OGTT was begun 24 hours after exercise, then there was no difference in glucose tolerance between the control and exercise condition.
Wheeler 2020 (29)	30’ Moderate bout<br><br>30’ Moderate bout and also adding breaks	Glucose tolerance sig worse after EX<br><br>Glucose tolerance sig worse after EX + breaks vs. prolonged sitting	A 30’moderaate intensity exercise bout significantly increased the total glucose AUC over 7.5 hrs (two test meals). This study demonstrated that when a walking bout ends in the postprandial period, the glucose can increase in the time after stopping a walking bout.<br><br>Adding 3’ walk breaks every 30’ over the 7 hours post exercise period did not improve the glucose tolerance.
Malin 2016 (45)	Moderate or high intensity	No effect on glucose tolerance	75 g OGTT was begun 1 hour after moderate or high intensity aerobic exercise at a duration to expend the same 200 kcal. Neither intensity improved the postprandial glucose response.
Knudsen 2014 (4)	1 hour at 50% VO2max, moderate intensity	NGT: higher<br>IGT: No effect<br>T2D: No effect	A key study that we describe in more detail in the text. The 75 g OGTT was begun after exercise and testing continued for 3 hours. Exercise raised postprandial glucose for NGT group and no effect on the glucose concentration at any time point in either the IGT or T2D groups. All three groups had an increase the rate of exogenous glucose appearance.
Holmstrup 2014 (39)	1 hour at 65% VO2max	Glucose tolerance sig worse after EX	Obese adults with impaired glucose tolerance (IGT). Carbohydrate was administered multiple times (every 2 hours) over a 12 hour period to sustain the postprandial period. From this repeated challenge approach, the exercise significantly increased the rise in the total response (12 hour iAUC) compared to the time matched sedentary control trial.
Rabol 2011 (50)	45’ at 75-85% HRmax	No effect on glucose tolerance	There was no improvement or worsening of glucose tolerance when the postprandial testing started 4.5 hours after finishing exercise bout. A mixed meal test was used after this relatively intense exercise.
Derave 2007 (51)	45’ at 60% VO2max	No effect on glucose tolerance	No effect of this exercise bout on postprandial glucose responses to a meal given 20’ after exercise stopped in sedentary men with metabolic syndrome
Rose 2001 (2)	55’ at 70% VO2max	Glucose tolerance sig worse after EX	75 g OGTT given 30’ after exercise ended. The prior exercise caused a remarkably 71% greater glucose iAUC than the OGTT without preceding exercise. This was in endurance trained participants.
Hamilton 1996 (1)	2.5 hours moderate treadmill	Glucose tolerance sig worse after EX	A mechanistic study highlighted in the text because duodenal glucose infusion was performed after exercise in surgically catheterized dogs to directly measure the absorption of exogenous glucose into the blood. The exercise increased the mixed venous blood glucose concentration by 15 mg/dL higher compared to control conditions while also increasing the rate of intestinal absorption.
Krzentowski 1982 (5)	3 hours at 50% VO2max	Glucose tolerance sig worse after EX	100 g OGTT given 30’ after exercise ended. There was a remarkable 60-70 mg/dL higher glucose in the 2nd hour of OGTT caused by prior exercise. Postprandial glucose oxidation was decreased and fat oxidation was increased throughout the whole ~7 hours after exercise stopped.

MISCELLANEOUS TYPES OF MUSCULAR ACTIVITY PERFORMED TO REDUCE SEDENTARY TIME WHEN REMAINING SEATED (i.e. NOT USING EITHER STANDING OR AMBULATORY BREAKS)			
Study	Activity	glucose Δ mg/dL	Annotated Comments
Han 2018 (48)	120' continuous low intensity cycling	0 NS	Overweight and obese sedentary office workers, most with prediabetes. Prolonged cycling did not cause a change in glucose tolerance in either men or women. Cycling power averaged ~40 watts. Although the VO_2 was not reported, we estimate from (from American College of Sports Medicine equations) the intensity of this large muscle mass activity was approximately 2.5-3 METs.
Petitt-Mee 2021 (46)	Very frequent "fidgeting" for 180'	-7*	A very rapid and very low range of motion bouncing action of the upper and lower legs ("fidgeting" at ~250 times per minute) was performed in obese participants. This was repeated for 2.5 min every 5 min with both limbs throughout the 3 hour OGTT. This rapid fidgeting increased the VO_2 by ~20% over the sitting inactive trial.
Hamilton 2022 (49)	SPU contractions continuously to target the soleus at two levels: +50% and +100% total body VO_2	-25*<br>-36*	At the more full level of this isolated contractile activity (+100% increase in total body VO2, which increased total body carbohydrate oxidation ~3X above sitting inactive), the average glucose concentration over the postprandial period was reduced by 36 mg/dL. In this time, the total 180 min glucose excursion (iAUC) was lowered by 52%, with the effect appearing quickly in the first phase while glucose was still rising and then sustained throughout the peak and beyond (i.e., a significant lowering of -19 mg/dL by 30 min, -28 mg/dL by 45 min, -43-49 mg/dL between 60-135', and -29-39 mg/dL between 150-180 min). These glucose responses were despite there being 60% less insulin concentration during SPU contractions. The C-peptide was also significantly reduced, indicative of less insulin secretion secondary to the lower rise in blood glucose. EMG, angular range of motion and velocity of the ankle joint, and the percent rise in VO_2 were used for individualized instruction. A dose-response analysis was made possible by using this randomized cross over study design. Results showed that there was a significant dose-response relationship between the metabolic intensity with an integrated index of glucose combined with insulin (and each outcome analyzed alone).
Elek 2025 (47)	120' continuous soleus contractions at 2 levels (VO2 not measured, but used EMG)	-24*<br>-32*	This was the first published confirmation of the original SPU study demonstrating improved glucose tolerance. Participants with prediabetes had a significant lowering of hyperglycemia during a 2 hour OGTT. Two trials with SPUs, but in neither was the actual metabolic intensity and carbohydrate oxidation measured. Although the ROM and VO2 was not monitored to optimize the SPU movement, they did demonstrate that the average glucose throughout the 2 hours was reduced by -32 mg/dL when soleus EMG feedback was provided, and by -24 mg/dL without their EMG measurements.

Supplementary Table Legend.

Glucose Δ, mg/dL is the time-averaged glucose difference between the sedentary control trial (acute sitting) and the physical activity intervention trial during the postprandial period. This effect is calculated by taking the difference in either the reported iAUC or AUC between trials and then dividing by the duration of the reported iAUC or AUC to yield an average glucose difference between trials. *denotes when the publication reported a P-value < 0.05 for the reported differences in postprandial glucose (iAUC or AUC or time-averaged glucose) between the activity intervention condition and its sitting inactive control condition. NS, reported to be not statistically significant. iAUC = incremental area under the curve above the fasting glucose value, AUC = total area under the curve for glucose. This table includes the "sedentary behavior" standing and brief break studies focused on glucose response reported in the most recent systematic review (Loh et al., Sports Medicine 2020; https://doi.org/10.1007/s40279-019-01183-w) cited in the recent large comprehensive narrative review (96) and any other missing references that we found in our literature review when writing the present article.
